# DNA Methylation and Its Role in Personalized Nutrition: Mechanisms, Clinical Insights, and Future Perspectives

**DOI:** 10.3390/ijms27020566

**Published:** 2026-01-06

**Authors:** Syed Ammar Hussain, Majher I. Sarker, Yanhong Liu, Tony Z. Jin

**Affiliations:** Eastern Regional Research Center, Agricultural Research Service, U.S. Department of Agriculture, 600 E. Mermaid Lane, Wyndmoor, PA 19038, USA; syed.a.hussain@usda.gov (S.A.H.); majher.sarker@usda.gov (M.I.S.); tony.jin@usda.gov (T.Z.J.)

**Keywords:** DNA methylation, personalized nutrition, one-carbon metabolism, epigenetic clocks, nutrigenomics, nutriepigenetics, dietary patterns, polyphenols, methyl donors, genotype-diet interactions, multi-omics, systems biology, biological aging, microbiome–epigenome interactions, precision nutrition

## Abstract

DNA methylation is a central epigenetic mechanism that mediates the interaction between nutritional exposures and gene regulation. Emerging evidence demonstrates that diet, bioactive compounds, genetic background, and lifestyle factors collectively shape the human methylome, influencing metabolic function, disease susceptibility, and biological aging. This review synthesizes current knowledge on the molecular and biochemical mechanisms of DNA methylation, the role of nutrients and dietary patterns in modulating methylation dynamics, and findings from human clinical trials evaluating nutritional interventions. Genotype-specific responses, including polymorphisms in one-carbon metabolism and metabolic pathways, are discussed as key determinants of interindividual variation in methylation outcomes. The review further highlights the advances in epigenetic clocks, systems biology, and multi-omics integration that support the development of precision nutrition frameworks. Ethical considerations and future challenges related to data interpretation, accessibility, and the regulation of epigenetic testing are also examined. Collectively, this review provides an integrative perspective on how DNA methylation serves as a dynamic interface between diet and health and outlines opportunities for implementing personalized nutrition strategies to improve metabolic resilience and promote healthy aging.

## 1. Introduction

Epigenetics refers to heritable yet reversible modifications to chromatin that regulate gene expression without altering the underlying DNA sequence. Among these mechanisms, including histone acetylation, histone methylation, RNA modifications, and chromatin remodeling, DNA methylation remains the most extensively studied due to its stability and central role in epigenetic inheritance. DNA methylation typically involves the enzymatic addition of a methyl group to the 5-carbon of cytosine residues to produce 5-methylcytosine, predominantly at CpG dinucleotides. These methylation marks regulate gene expression by influencing transcription factor binding, modulating chromatin accessibility, and recruiting methyl-binding proteins that contribute to chromatin compaction [1,2,3].

Aberrant DNA methylation patterns have been associated with a broad range of diseases, including cancer, metabolic syndrome, obesity, type 2 diabetes, neurodegenerative disorders, cardiovascular disease, and immune dysfunction [4,5,6]. Global hypomethylation contributes to chromosomal instability and activation of transposable elements, while hypermethylation of tumor suppressor genes results in transcriptional silencing. DNA methylation patterns also change progressively with age through a process known as epigenetic drift, which reflects stochastic methylation alterations driven by environmental exposures, lifestyle factors, and accumulated molecular damage [7,8].

Nutrition is one of the most influential environmental factors shaping the methylome. Nutrients involved in one-carbon metabolism, such as folate, vitamin B12, vitamin B6, choline, betaine, and methionine, supply the methyl groups required to produce S-adenosylmethionine (SAM), the universal methyl donor for DNA, RNA, and histone methylation reactions. Disruptions in the availability of methyl donors can impair methylation fidelity, contributing to transcriptional dysregulation and metabolic disturbances [9,10,11]. Classic examples include folate deficiency leading to neural tube defects and global hypomethylation, as well as maternal choline intake affecting offspring neurodevelopment and hippocampal DNA methylation patterns [12,13].

Beyond essential nutrients, several bioactive dietary compounds including polyphenols such as epigallocatechin gallate, curcumin, resveratrol, and genistein, as well as isothiocyanates, short-chain fatty acids, and carotenoids have been shown to influence DNA methylation. These compounds may inhibit DNA methyltransferases, modulate TET enzyme activity, or alter chromatin structure, thereby affecting gene expression and long-term metabolic programming [14,15]. Such findings highlight diet as a powerful modulator of epigenetic regulation.

Recent advances in nutrigenomics and nutriepigenomics have demonstrated that individual genetic background interacts with nutrient intake to shape epigenetic outcomes. Polymorphisms in key one-carbon metabolism genes, including MTHFR C677T, MTRR, BHMT, and CBS, influence methylation efficiency and contribute to inter-individual variability in response to folate or methyl donor supplementation [15,16]. These genotype-specific differences form an important basis for personalized nutrition, which aims to optimize dietary recommendations based on an individual’s genomic and epigenomic profile.

The emergence of epigenetic clocks, which estimate biological age based on methylation signatures at selected CpG sites, has further advanced the integration of DNA methylation into nutrition research. Dietary interventions such as caloric restriction, increased polyphenol intake, vitamin D supplementation, plant-based diets, and methyl donor–rich diets have been associated with favorable shifts in epigenetic aging markers [17,18]. These findings suggest that nutrition can modulate biological aging trajectories. Parallel advancements in multi-omics integration, machine learning, and artificial intelligence have enabled the analysis of complex interactions among genetic, epigenetic, transcriptomic, microbiome, dietary, and metabolic factors. These tools enhance the predictive capacity of personalized nutrition frameworks and support the development of precision interventions for disease prevention and health optimization [16,17,18,19].

The aim of this review is to synthesize current evidence on the role of DNA methylation in personalized nutrition. This includes an overview of the molecular mechanisms underlying DNA methylation, dietary and lifestyle influences on methylation patterns, findings from human intervention trials, and insights into genotype-guided dietary strategies. The review also examines epigenetic clocks, multi-omics integration, tissue-specific methylation challenges, advances in methylation profiling technologies, and ethical considerations relevant to the implementation of epigenetic biomarkers in precision nutrition. By integrating mechanistic, clinical, and technological perspectives, this review highlights the role of DNA methylation as a dynamic interface between diet and gene regulation, providing a foundation for personalized and preventive nutrition approaches.

## 2. Molecular Mechanisms of DNA Methylation

DNA methylation is a fundamental epigenetic modification that regulates gene expression, chromatin structure, and genomic stability. In mammals, DNA methylation primarily occurs at cytosine residues within CpG dinucleotides, producing 5-methylcytosine. This modification is established and maintained by a family of DNA methyltransferases (DNMTs), and its dynamic regulation is influenced by nutrient availability, cellular metabolism, and environmental factors.

DNMT1 is the primary maintenance methyltransferase and preferentially methylates hemi-methylated DNA during replication, ensuring that methylation patterns are preserved in daughter cells. DNMT3A and DNMT3B function mainly as de novo methyltransferases, establishing new methylation marks during embryonic development and cellular differentiation. DNMT3L, although catalytically inactive, acts as a regulatory cofactor that enhances DNMT3A and DNMT3B activity, particularly in germ cells. Disruptions to DNMT activity can lead to global hypomethylation, gene-specific hypermethylation, and widespread transcriptional dysregulation [20,21].

The methylation process requires a continuous supply of methyl groups, which are donated by S-adenosylmethionine (SAM). SAM is synthesized through one-carbon metabolism, a network of biochemical reactions involving folate, vitamin B12, vitamin B6, choline, betaine, and methionine. These nutrients regulate the balance between SAM and S-adenosylhomocysteine (SAH), a potent inhibitor of DNMTs. A low SAM:SAH ratio reduces methylation potential and promotes hypomethylation, highlighting the strong dependence of DNA methylation on nutritional status and metabolic flux [22,23].

CpG sites vary in their distribution and regulatory function. CpG islands, which are enriched in CpG dinucleotides, are commonly located in gene promoters and are typically unmethylated to permit transcription. Aberrant hypermethylation of CpG islands can silence tumor suppressor genes, contributing to carcinogenesis. CpG shores and shelves, regions adjacent to CpG islands, exhibit greater variability and appear more responsive to environmental influences, including diet. Non-CpG methylation (CpA, CpT, CpC), although less common, is prominent in embryonic stem cells and neural tissues and contributes to development and neuronal plasticity [20,21,22,23].

DNA methylation influences gene expression through multiple mechanisms. Methylation can directly block the binding of transcription factors to DNA or promote the recruitment of methyl-binding proteins such as MeCP2 and MBD family proteins. These proteins interact with histone deacetylases and other chromatin-modifying complexes, resulting in chromatin compaction and transcriptional repression. Thus, DNA methylation acts in coordination with histone modifications and nucleosome positioning to regulate gene activity [11,12].

Although once considered irreversible, DNA methylation is now understood to be dynamically regulated. Active demethylation is mediated by the ten-eleven translocation (TET) family of enzymes, which oxidize 5-methylcytosine to 5-hydroxymethylcytosine and further oxidize the intermediates. These modified bases can be replaced with unmethylated cytosine through base excision repair. TET enzyme activity depends on cofactors such as iron and α-ketoglutarate and can be influenced by vitamin C, cellular redox state, and mitochondrial metabolism [14,22].

With aging, DNA methylation patterns become increasingly disordered through a process known as epigenetic drift. This includes global hypomethylation, localized hypermethylation, and increased variability among cells. Epigenetic drift reflects cumulative environmental exposures, inflammation, metabolic stress, and lifestyle factors. These age-associated methylation changes form the basis for epigenetic clocks, which estimate biological age and have emerged as valuable biomarkers for studying the impact of diet and lifestyle on aging processes [15,23].

**Figure 1:**
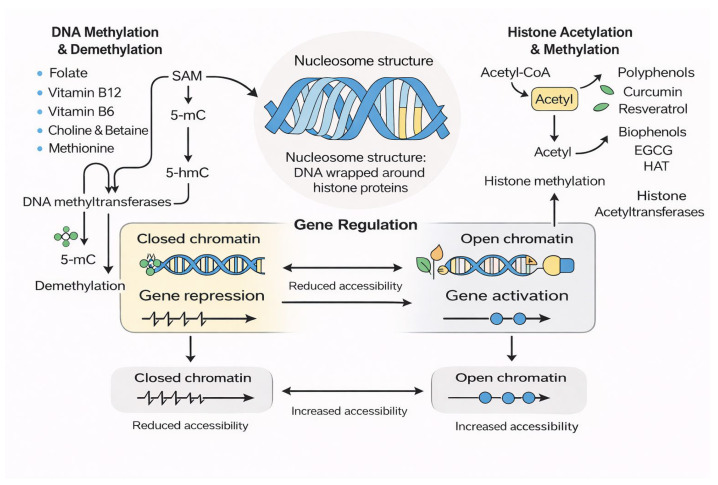
Nutrient-Mediated Regulation of DNA and Histone Modifications.

The methylome is sensitive to environmental and nutritional influences throughout life. Dietary methyl donors, polyphenols, microbiome-derived metabolites, physical activity, psychological stress, alcohol consumption, smoking, and exposure to environmental toxins can all influence DNA methylation. Some genomic regions, known as metastable epialleles, are especially responsive to early-life nutritional environments and can shape long-term health outcomes. This sensitivity underscores the importance of understanding how molecular methylation pathways respond to dietary patterns and lifestyle factors [13,24]. These enzymatic interactions, together with nutrient-derived one-carbon metabolism and chromatin-modifying pathways, are illustrated schematically in Figure 1.

## 3. Dietary Modulation of DNA Methylation

Diet plays a major role in shaping DNA methylation patterns because many nutrients directly influence one-carbon metabolism and methyl group availability. In addition to classical methyl-donor nutrients, numerous bioactive dietary compounds can modulate DNA methyltransferase activity, alter chromatin accessibility, or influence metabolic pathways. As a result, nutrition is one of the most significant environmental determinants of the human methylome across the lifespan.

### 3.1. Methyl Donors and One-Carbon Metabolism

Dietary methyl donors such as folate, vitamin B12, vitamin B6, choline, betaine, and methionine influence DNA methylation primarily by modulating one-carbon metabolism and cellular methylation potential, as described in Section 2. Variations in methyl donor availability affect the SAM:SAH ratio and thereby regulate global and gene-specific methylation capacity [24,25,26]. Deficiencies in folate, vitamin B12, or other methyl donors can alter methylation homeostasis. Low folate intake has been associated with global hypomethylation and increased risk of neural tube defects and colorectal cancer [26,27]. Vitamin B12 deficiency increases homocysteine concentrations and reduces methylation potential. Choline and betaine provide alternative pathways for homocysteine remethylation, and insufficient intake of these nutrients has been linked to hepatic lipid accumulation and altered gene-specific methylation patterns in both human and animal studies [9,10,11,12,13,14,15,16,17,18]. These findings highlight the dependence of DNA methylation on dietary methyl-donor availability.

### 3.2. Polyphenols and Other Bioactive Dietary Compounds

Polyphenols are abundant dietary molecules capable of modifying epigenetic regulation. Several well-studied polyphenols including epigallocatechin gallate (EGCG), genistein, curcumin, and resveratrol have demonstrated effects on DNA methylation by inhibiting DNMT activity, modulating TET enzymes, and influencing chromatin conformation [28,29,30]. EGCG, a major green tea catechin, can inhibit DNMT1 and reactivate silenced tumor suppressor genes in vitro. Genistein from soy has shown gene-specific methylation changes associated with cancer prevention and metabolic regulation. Curcumin and resveratrol exhibit similar epigenetic effects, acting through DNMT inhibition and modulation of histone-modifying enzymes [31]. Other dietary bioactive also influence DNA methylation. Sulforaphane, found in cruciferous vegetables, affects both DNMT and histone deacetylase activity and contributes to chemopreventive effects. Short-chain fatty acids (SCFAs), particularly butyrate produced by microbial fermentation of dietary fiber, function as histone deacetylase inhibitors and indirectly influence methylation through altered chromatin accessibility [30]. Carotenoids, omega-3 fatty acids, and antioxidants may further modulate methylation via effects on oxidative stress and inflammatory signaling pathways [29,30]. These compounds illustrate how diverse dietary components participate in epigenetic regulation. Several dietary compounds have been shown to modulate the epigenetic state of specific tumor suppressor genes through changes in promoter methylation. For example, epigallocatechin gallate (EGCG), a major catechin in green tea, can reduce promoter hypermethylation of p16INK4a and RASSF1A in breast and prostate epithelial cells, leading to partial reactivation of these genes and restoration of cell-cycle regulation. Similar effects have been reported for curcumin and genistein, which can demethylate promoter regions of PTEN and BRCA1 in breast cancer cell models, thereby influencing pathways involved in apoptosis and DNA repair. These findings illustrate how nutrients and bioactive dietary compounds can directly alter the methylation status of tumor suppressor genes in relevant tissues, providing a mechanistic basis for their potential role in disease prevention [28,29,30,31].

### 3.3. Dietary Patterns and Epigenetic Responses

Beyond individual nutrients, whole dietary patterns have been shown to influence DNA methylation profiles. Diets rich in fruits, vegetables, legumes, whole grains, and plant-based foods such as plant-forward diets and Mediterranean-style dietary patterns provide methyl donors, polyphenols, and antioxidant compounds that collectively support epigenetic stability [19]. These dietary patterns have been associated with favorable changes in inflammatory markers, metabolic regulation, and biological aging indicators partly through epigenetic mechanisms.

In contrast, Western dietary patterns high in processed foods, saturated fats, and refined carbohydrates have been linked to adverse methylation signatures, including hypermethylation of genes involved in metabolic regulation and increased risk for obesity and insulin resistance [20]. Human intervention studies indicate that diet-induced methylation changes can occur within weeks and, in some cases, may be reversible. Short-term interventions involving vegan diets, caloric restriction, or omega-3 fatty acid supplementation have demonstrated alterations in methylation profiles consistent with improved metabolic and inflammatory outcomes [21,22,23].

### 3.4. Short-Term and Long-Term Dietary Effects on the Methylome

The timing and persistence of diet-induced methylation changes vary substantially. Short-term dietary modifications can rapidly alter methylation at genes involved in metabolism, inflammation, and oxidative stress responses. Long-term dietary habits, particularly during early development, have more persistent effects and may establish stable epigenetic signatures associated with lifelong disease risk [24].

Maternal nutrient intake including folate, choline, and methionine, affects fetal methylation patterns and influences neurodevelopment, immune function, and metabolic health later in life [25,26]. Early-life epigenetic responsiveness is exemplified by metastable epialleles, genomic regions particularly sensitive to nutritional conditions during critical developmental windows. Long-term adherence to nutrient-rich dietary patterns has also been associated with slower epigenetic aging, whereas sustained exposure to unhealthy dietary patterns accelerates biological age as measured by DNA methylation clocks [27,28]. Collectively, these findings demonstrate that diet is a potent modulator of the human methylome and contributes to both short-term metabolic regulation and long-term health trajectories.

## 4. Human Clinical Trials and Nutritional Interventions

Human clinical studies provide essential evidence linking dietary exposures to DNA methylation changes and associated health outcomes. While mechanistic and animal studies have established strong biological plausibility for nutrient–methylation interactions, clinical trials help determine whether these effects occur in human populations and whether they translate into improvements in metabolic, inflammatory, or aging-related biomarkers. Recent trials have examined the effects of methyl donor supplementation, polyphenol-rich diets, vegan versus omnivorous dietary patterns, omega-3 fatty acid intake, vitamin D supplementation, and fermented foods on DNA methylation and epigenetic aging [32,33].

### 4.1. Methyl Donor Supplementation and One-Carbon Nutrient Trials

Building on the mechanistic framework of one-carbon metabolism outlined in Section 2, multiple clinical trials have examined whether methyl donor supplementation translates into measurable changes in DNA methylation in humans. Supplementation with folic acid has been shown to increase global methylation levels, particularly LINE-1 repetitive element methylation, in populations at risk for folate deficiency [34,35]. Some studies have also reported gene-specific methylation changes in pathways related to inflammation, lipid metabolism, and carcinogenesis following folic acid intake.

Vitamin B12 supplementation has demonstrated similar but more variable effects, partly because of inter-individual differences in baseline B12 status, absorption rates, and genetic polymorphisms in one-carbon metabolism genes. Individuals with the MTHFR C677T polymorphism often show greater responsiveness to folate interventions, highlighting the importance of genetic context in determining methylation outcomes [36,37]. Choline and betaine supplementation studies have shown modest improvements in hepatic methylation patterns and reductions in homocysteine levels, indicating their potential role in metabolic health and liver function [38,39].

### 4.2. Polyphenol-Rich Diets and DNA Methylation Changes

Polyphenols modulate DNA methylation through mechanisms described in Section 3.2, including DNMT inhibition and altered chromatin accessibility. Clinical studies therefore primarily assess whether these molecular effects translate into reproducible methylation changes in vivo. Green tea catechins, particularly epigallocatechin gallate (EGCG), have been associated with DNMT inhibition and modifications to promote methylation in genes related to tumor suppression and oxidative stress responses [9]. Soy isoflavone supplementation has been linked to changes in methylation of genes involved in estrogen signaling and inflammation [10]. Clinical trials exploring curcumin and resveratrol supplementation have reported small but measurable shifts in methylation of metabolic and inflammatory genes, although the magnitude of changes is often modest and varies across studies [11,12]. Overall, polyphenol interventions demonstrate that dietary bioactive compounds can influence methylation patterns in humans, but the variability in dose, duration, bioavailability, and study populations complicates direct comparisons.

### 4.3. Diet, Epigenetic Aging, and DNA Methylation Clocks

A growing body of clinical evidence examines how diet influences DNA methylation–based biological aging estimates. Epigenetic clocks, including Horvath, Hannum, GrimAge, and PhenoAge, provide biomarkers of biological age acceleration or deceleration. Several trials have reported that specific dietary interventions modestly slow epigenetic aging. Vitamin D and omega-3 fatty acid supplementation in older adults has been associated with reduced biological age acceleration in large clinical studies, including the DO-HEALTH trial [13]. Plant-based and vegan dietary interventions have shown similar effects; in one study involving monozygotic twins, individuals adhering to a vegan diet exhibited younger predicted epigenetic ages over a short intervention period compared with their omnivorous co-twins [14]. Caloric restriction trials have also demonstrated beneficial effects on methylation at age-related CpG sites, suggesting diet-related modulation of aging pathways [15]. These findings indicate that dietary composition can affect epigenetic aging trajectories, although more long-term studies are needed to establish sustained effects.

### 4.4. Fermented Foods, Gut Microbiota, and Epigenetic Responses

Fermented foods have gained interest in their ability to modulate gut microbiota composition, reduce inflammation, and influence host metabolic pathways. Recent clinical studies show that fermented vegetable intake can alter DNA methylation in genes involved in lipid metabolism, immune regulation, and liver function [16]. Individuals carrying risk alleles in PNPLA3, a gene associated with non-alcoholic fatty liver disease, appear particularly responsive to fermented food interventions, suggesting gene–diet interactions in epigenetic outcomes [17]. Short-chain fatty acids such as butyrate produced through microbial fermentation of dietary fiber also contribute to epigenetic regulation by functioning as histone deacetylase inhibitors and indirectly influencing DNA methylation through modifications in chromatin accessibility [18]. These findings highlight the interconnected roles of diet, microbiome metabolism, and epigenetic regulation.

### 4.5. Methodological Challenges in Nutritional Epigenetics Trials

Despite increasing interest in nutritional epigenetics, several methodological challenges complicate the interpretation of dietary intervention studies. A major limitation is cell-type heterogeneity in peripheral blood DNA methylation data. Because circulating immune cell populations vary with age, inflammation, circadian rhythm, and diet-induced metabolic changes, observed methylation differences may reflect shifts in cell composition rather than true locus-specific epigenetic modifications [19,40]. Although statistical deconvolution methods have been developed to estimate cell-type proportions, these approaches rely on reference datasets and modeling assumptions that can introduce additional uncertainty and affect reproducibility across studies [41].

Another important challenge involves batch effects and cross-platform inconsistencies. DNA methylation measurements generated using Illumina 450K arrays, EPIC arrays, whole-genome bisulfite sequencing, or targeted amplicon-based approaches are not always directly comparable. Differences in probe design, genomic coverage, hybridization chemistry, normalization pipelines, and bioinformatic processing can introduce platform-specific signatures that complicate cross-study replication and meta-analysis [42,43].

Replication is further hindered by the small sample sizes typical of many nutritional intervention trials. Epigenetic responses to dietary exposures are often modest, tissue dependent, and influenced by numerous covariates, including age, sex, baseline metabolic status, and lifestyle factors. As a result, underpowered studies may fail to detect biologically meaningful changes or may yield unstable effect estimates [44]. Longitudinal study designs introduce additional complexities, including participant dropouts, behavioral variability, and reliance on relatively short intervention periods that may be insufficient to capture durable or cumulative DNA methylation changes [45].

A further complication arises from confounding between weight loss and diet composition, particularly in dietary aging or anti-aging studies. Caloric restriction, improvements in metabolic health, and reductions in adiposity independently influence DNA methylation patterns and epigenetic aging metrics, making it difficult to isolate the specific contribution of nutrient composition or individual bioactive compounds [46,47]. Without careful control for weight change, attribution of observed methylation differences to the dietary intervention itself may be inaccurate.

Finally, nutritional epigenetics studies must contend with substantial inter-individual variability driven by genetic background, baseline methylation state, microbiome composition, sleep patterns, psychosocial stress, and physical activity. These factors interact with dietary exposures and may contribute to inconsistent findings across studies [48,49]. Addressing these methodological limitations through larger and more diverse cohorts, harmonized measurement platforms, rigorous control of confounders, and standardized analytical pipelines will be essential for improving reproducibility and translating nutritional epigenetics into clinical and public health applications.

### 4.6. Summary of Clinical Evidence

Overall, the available clinical evidence supports the notion that diet can influence DNA methylation in humans and that targeted nutritional interventions may promote metabolic health, modulate inflammatory pathways, and slow biological aging. The most promising effects have been observed in methyl donor supplementation for individuals with deficiencies or genetic variants affecting one-carbon metabolism, in polyphenol-rich dietary interventions, and in dietary patterns associated with healthier metabolic profiles. Future studies should integrate personalized approaches that consider individual genetic backgrounds, baseline methylation patterns, and microbiome profiles to enhance the precision and effectiveness of nutritional epigenetic strategies.

## 5. Genotype-Specific Responses and Personalized Nutrition

Genetic variability plays a critical role in determining individual responses to dietary components and influences the extent to which specific nutrients modify DNA methylation patterns. Polymorphisms in genes regulating one-carbon metabolism, methyl donor utilization, and nutrient transport can alter methylation efficiency and contribute to inter-individual differences in metabolic outcomes. Understanding these genotype–nutrition–methylation interactions is essential for developing effective personalized nutrition strategies aimed at disease prevention and metabolic optimization [1,2,3].

### 5.1. Genetic Variation in One-Carbon Metabolism Genes

One-carbon metabolism relies on the coordinated activity of several enzymes to generate and recycle methyl groups used for DNA and histone methylation. Polymorphisms in these enzymes affect methylation capacity and influence susceptibility to nutrient deficiencies. One of the most studied variants is the MTHFR C677T polymorphism, which reduces enzyme activity and lowers the conversion of 5,10-methylenetetrahydrofolate to 5-methyltetrahydrofolate. Individuals carrying the TT genotype often exhibit elevated homocysteine levels, reduced methylation potential, and increased sensitivity to folate intake [4,5]. Clinical studies demonstrate that individuals with MTHFR C677T variants show greater changes in DNA methylation following folate supplementation compared with CC genotype carriers, indicating that genetic background modulates nutritional responsiveness [6]. Other key genes influencing methylation capacity include MTR, MTRR, BHMT, CBS, and CHDH. Variants within these genes can disrupt homocysteine remethylation, alter methyl donor availability, and contribute to changes in global or gene-specific methylation profiles [7,8,9]. For example, polymorphisms in BHMT have been associated with altered choline metabolism, while CBS variants affect homocysteine transculturation. These genetic influences underscore the importance of considering individual polymorphisms when evaluating dietary effects on the methylome.

### 5.2. DNA Methylation Sensitivity to Macronutrient Ratios

Emerging evidence suggests that genotypes can influence metabolic responses to dietary macronutrient composition, including carbohydrate, fat, and protein intake. Variants in genes related to insulin signaling, lipid metabolism, and adipogenesis such as FTO, TCF7L2, APOA5, and PPARγ have been associated with distinct epigenetic responses to dietary macronutrients [10,11]. Individuals with risk alleles in FTO may experience exaggerated methylation changes in appetite-regulating pathways following high-fat or high-calorie meals. Similarly, carriers of TCF7L2 variants, which confer increased risk of type 2 diabetes, exhibit altered methylation responses to carbohydrate-rich meals, affecting glucose metabolism and insulin secretion [12]. Studies suggest that dietary macronutrient ratios may interact with genetic background to influence epigenetic regulation of metabolic pathways, although more controlled trials are needed to fully clarify these relationships. Although genotype diet interactions provide compelling hypotheses for personalized nutrition, most findings remain correlational and derive from observational datasets. Sample sizes are often limited, and gene-environment interactions can be confounded by metabolic, behavioral, and demographic factors. Therefore, these associations should be interpreted cautiously until validated in larger, controlled intervention studies.

### 5.3. Nutrigenetic Modulation of Polyphenol and Bioactive Compound Responses

Genetic variation may also influence responsiveness to polyphenols and other bioactive dietary components. For instance, polymorphisms in genes encoding detoxification enzymes, such as GSTM1, GSTT1, and COMT affect the metabolism and bioavailability of polyphenols, contributing to inter-individual variability in epigenetic outcomes [13]. Individuals with specific COMT variants may experience stronger methylation changes in inflammatory or metabolic genes following intake of green tea catechins or soy isoflavones. Similarly, TET enzyme–related polymorphisms may influence the degree to which individuals respond to vitamin C, alpha-ketoglutarate, and antioxidant-rich foods that modulate active demethylation pathways [14]. These genotype-specific differences highlight the potential for tailoring polyphenol-based dietary interventions to optimize epigenetic benefits. Evidence for interindividual variability in epigenetic responses to diet is growing, but much of the current literature relies on cross-sectional data or heterogeneous study designs. Differences in diet assessment, methylation platforms, and population characteristics may contribute to inconsistent findings. Longitudinal studies and controlled feeding trials are needed to clarify causality and refine personalized nutrition approaches.

### 5.4. Gene–Diet Interactions in Chronic Disease Risk

Personalized nutrition has significant implications for chronic disease prevention. Gene–diet interactions have been documented in obesity, cardiovascular disease, diabetes, cancer susceptibility, and neurodegenerative disorders. For example, carriers of APOE ε4 alleles show distinct methylation and metabolic responses to dietary fat quality, with implications for lipid metabolism and cardiovascular risk. Individuals with PNPLA3 I148M variants, associated with non-alcoholic fatty liver disease, demonstrate differential methylation responses to dietary choline and fermented foods, suggesting that epigenetic regulation may mediate gene–diet effects on liver health [15,16]. In colorectal cancer, variants in folate metabolism genes modify the relationship between dietary folate intake, DNA methylation, and tumor risk [17]. Such findings indicate that personalized dietary strategies based on genetic markers may enhance the efficacy of nutritional interventions in disease prevention. Although mechanistic links between microbial metabolites and DNA methylation are biologically plausible, human evidence remains preliminary. Many studies infer associations from stool microbiome profiles and peripheral blood methylation, which may not capture tissue-specific mechanisms. More rigorously controlled trials integrating microbiome, metabolomic, and epigenomic data are required to establish causality.

### 5.5. Integrating Genetic and Epigenetic Biomarkers in Personalized Nutrition

The combined use of genetic variants and DNA methylation biomarkers provides a targeted, biomarker-driven foundation for personalized nutrition. Genetic information offers relatively stable insight into inherited predispositions affecting nutrient metabolism, absorption, and utilization, whereas DNA methylation captures dynamic, exposure-responsive signals reflecting diet, lifestyle, and environmental influences [50,51]. Integrating these two layers enables refined stratification of individuals based on both biological predisposition and current molecular state, improving the prediction of dietary responsiveness, metabolic risk, and intervention outcomes.

**Figure 2:**
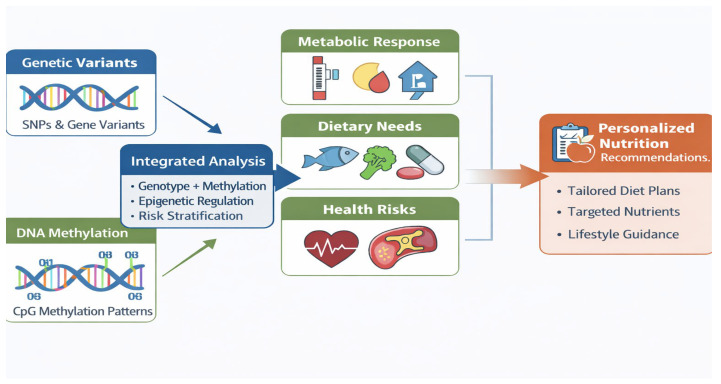
Integration of genetic variants and DNA methylation biomarkers to inform personalized nutrition. Genetic predisposition (single-nucleotide polymorphisms) and CpG methylation patterns are jointly analyzed to stratify metabolic responses, dietary needs, and health risks, enabling tailored nutritional recommendations.

In this framework, DNA methylation functions as an intermediate phenotype linking genotype to functional outcomes such as altered gene expression, pathway activity, and metabolic regulation [10]. Several studies integrating genetic variants with methylation markers have demonstrated improved prediction of postprandial glycemic responses, lipid metabolism, and weight-loss success compared with models relying on genetic data alone [18,19,20,35]. Notably, these approaches often focus on defined sets of nutrients- and metabolism-relevant CpG sites or candidate loci rather than full systems-level integration, enhancing interpretability and translational potential. Figure 2 illustrates this biomarker-centric approach, in which genetic variants and DNA methylation markers are jointly leveraged to inform individualized dietary guidance.

Despite their promise, genotype–methylation models face important limitations. Outcomes can be influenced by population heterogeneity, methylation platform variability, normalization strategies, and modest sample sizes [52]. Moreover, many studies remain observational or correlational, underscoring the need for longitudinal designs and controlled dietary intervention trials to establish causal relationships. Nonetheless, integrating genetic and epigenetic biomarkers represents a critical step toward actionable personalized nutrition, providing a practical bridge between molecular mechanisms and individualized dietary decision-making.

### 5.6. Implications for Precision Nutrition and Public Health

Understanding genotype-specific methylation responses has significant implications for both precision nutrition and public health nutrition guidelines. While population-based dietary recommendations aim to promote general health, personalized approaches can target individuals most likely to benefit from specific nutrients or dietary patterns based on their genetic and epigenetic profiles. For example, individuals with reduced folate metabolism capacity may require higher folate or choline intake; those with altered lipid metabolism genes may benefit from specific fat compositions; and individuals genetically predisposed to accelerated epigenetic aging may benefit from diets rich in antioxidants, polyphenols, and methyl donors. Tailored dietary interventions have the potential to improve treatment outcomes, enhance metabolic resilience, and reduce chronic disease burden at the individual and population levels.

## 6. DNA Methylation and Aging: Insights from Epigenetic Clocks

**Figure 3:**
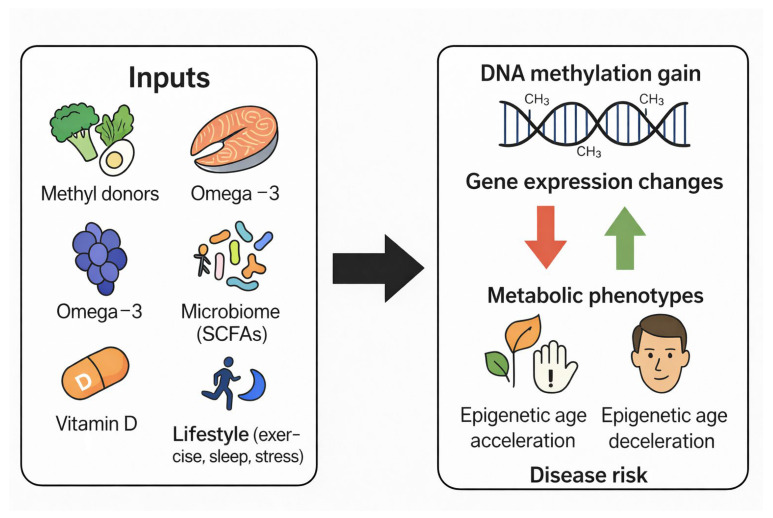
Dietary, Genetic, and Environmental Modulators of DNA Methylation and Their Effects on Health and Aging.

DNA methylation patterns change progressively with age and reflect underlying biological processes such as genomic instability, inflammation, mitochondrial dysfunction, and oxidative stress. These predictable, age-associated methylation changes have led to the development of epigenetic clocks, mathematical models that estimate biological age based on methylation levels at selected CpG sites. Epigenetic clocks provide a quantitative measure of biological aging that often correlates more strongly with health status, disease risk, and mortality than chronological age (Figure 3) [37,38,39,53,54,55,56,57,58,59,60,61].

### 6.1. Age-Associated DNA Methylation Drift

Age-associated epigenetic drift reflects the cumulative impact of stochastic methylation errors, environmental exposures, metabolic stress, and inflammatory signaling, as introduced in Section 2. These coordinated changes manifest as global hypomethylation, localized promoter hypermethylation, and increased inter-individual variability, forming the molecular basis for epigenetic clock development [62,63]. Global hypomethylation contributes to chromosomal instability and activation of transposable elements, whereas promoter hypermethylation can silence genes involved in DNA repair, cell cycle control, and tumor suppression. In addition to environmental influences, several intrinsic biological mechanisms contribute to age-associated alterations in DNA methylation patterns. One major driver is stochastic epigenetic drift, which reflects random, replication-associated errors in methylation maintenance that accumulate over time as DNMT1 fidelity decreases. Aging is also associated with reduced activity and altered targeting of DNMT3A/3B and TET enzymes, leading to both aberrant hypermethylation at CpG islands and global hypomethylation at repetitive elements. Chromatin remodeling becomes progressively dysregulated with age, resulting in altered nucleosome positioning, histone mark redistribution, and accessibility changes that influence local methylation patterns. Furthermore, chronic low-grade inflammation (“inflammaging”) promotes methylation reprogramming through sustained cytokine signaling, oxidative stress, and activation of immune-related transcriptional pathways. Mitochondrial dysfunction contributes additional metabolic constraints such as disruptions in α-ketoglutarate and NAD^+^ availability that affect TET-mediated demethylation. Finally, impaired DNA repair pathways in aging cells create altered methylation signatures at damage sites due to differential recruitment of DNMTs and chromatin modifiers. Together, these intrinsic processes interact with environmental exposures to shape the characteristic methylation landscape of aging tissues. Epigenetic drift is influenced by cumulative exposure to environmental factors such as diet, smoking, pollution, alcohol consumption, stress, and physical inactivity [64]. These exposures leave “epigenetic signatures” that accumulate over time, contributing to accelerated biological aging.

### 6.2. Development and Types of Epigenetic Clocks

Epigenetic clocks have evolved from first-generation predictors of chronological age (e.g., Horvath, Hannum) to second-generation models incorporating morbidity and mortality risk (e.g., PhenoAge, GrimAge), and more recently to dynamic measures of aging rate such as DunedinPACE. Collectively, these clocks quantify distinct but overlapping aspects of biological aging and are widely used to evaluate lifestyle and dietary influences on aging trajectories [65,66,67,68,69]. Although epigenetic clocks capture reproducible age-associated methylation changes across many tissues, it is important to recognize that the underlying biology is not uniform across the body. Epigenetic clocks are trained predominantly on CpG sites that show consistent directional changes with age across diverse tissues; however, several organs exhibit stable methylation profiles over the lifespan. Prior study demonstrated that portions of the human brain and other slowly renewing tissues show minimal or no methylation change with age, indicating that epigenetic aging signatures are highly tissue dependent. This highlights a fundamental limitation of pan-tissue epigenetic clocks, they capture systemic aging trends but may not fully reflect the aging trajectory of tissues with low proliferative activity or tightly regulated chromatin states. These tissue-specific constraints are important when interpreting dietary or lifestyle effects on epigenetic aging, as changes detected in peripheral blood or buccal cells may not generalize to all organ systems [8,48,69].

### 6.3. Dietary and Lifestyle Modulators of Epigenetic Aging

A growing body of research indicates that diet and lifestyle exert measurable effects on DNA methylation–based biological aging. Epigenetic clocks, that often correlate with health outcomes more strongly than chronological age. These tools have facilitated the study of how nutritional patterns, micronutrients, metabolic exposures, and behavioral factors influence aging trajectories.

#### 6.3.1. Dietary Patterns and Epigenetic Aging

Diets rich in polyphenols, antioxidants, methyl donors, and anti-inflammatory compounds such as plant-forward or Mediterranean-style diets have been associated with lower epigenetic age acceleration. Higher consumption of leafy greens, cruciferous vegetables, whole grains, and fiber correlates with more favorable methylation signatures at age-related CpG sites, whereas Western dietary patterns high in processed foods and saturated fat are linked to accelerated epigenetic aging [70,71,72,73]. Caloric restriction, intermittent fasting, and omega-3 fatty acid intake have demonstrated reductions in biological age acceleration, particularly at CpG sites associated with inflammation and metabolic regulation [73].

#### 6.3.2. Nutrient-Specific Effects on Aging Methylation Signatures

Several nutrients modulate methylation marks that contribute to biological age. Folate, vitamin B12, vitamin B6, choline, and methionine regulate methyl donor availability and influence methylation at aging-sensitive regions [74,75]. Vitamin D supplementation has been associated with slower epigenetic aging in older adults [76,77]. Polyphenols including EGCG, resveratrol, quercetin, and curcumin may influence aging via DNMT and TET modulation, antioxidant effects, and stabilization of methylation patterns [78,79].

#### 6.3.3. Interactions with Exercise, Sleep, and Stress

Lifestyle factors interact with dietary exposures to shape epigenetic aging. Regular physical activity is consistently linked to reduced epigenetic age acceleration, particularly in moderate-to-vigorous exercise cohorts [80]. Sleep quality and circadian rhythm alignment influence methylation at genes regulating inflammation and metabolic homeostasis [81]. Conversely, chronic psychological stress accelerates epigenetic aging through glucocorticoid-related methylation changes, especially in immune and neural pathways [82]. These findings support integrated lifestyle strategies for reducing age acceleration.

#### 6.3.4. Diet and Lifestyle Interventions Assessed by Epigenetic Clocks

Epigenetic clocks have been used to evaluate the impact of combined dietary and behavioral interventions. A multi-component diet–lifestyle program incorporating plant-rich foods, probiotics, exercise, and stress reduction resulted in a reduction of ~1–3 years in biological age within eight weeks [83]. Long-term caloric restriction trials similarly demonstrate reduced pace-of-aging estimates compared to controls [84]. Supplementation with omega-3 fatty acids and vitamin D has produced measurable improvements in methylation markers associated with inflammation and aging [85].

#### 6.3.5. Reversibility of Epigenetic Aging

Evidence suggests that biological aging, as assessed by DNA methylation clocks, is at least partially reversible. Nutritional interventions, lifestyle modifications, and targeted supplementation can shift methylation signatures toward more youthful patterns, although the durability and tissue specificity of these changes remain active areas of investigation [63,86]. Importantly, these intervention-driven methylation changes reflect physiological remodeling within intact tissues and should be distinguished from the global epigenetic resetting observed during induced pluripotent stem cell (iPSC) reprogramming, which is not related to lifestyle or nutritional influences. Understanding the mechanisms that enable such physiologically achievable reversibility will be critical for developing precision nutrition strategies to support long-term metabolic health and healthy aging.

Although numerous studies report associations between diet, lifestyle, and epigenetic aging, the strength of this evidence remains limited by several methodological constraints. Many findings derive from observational or cross-sectional designs that cannot establish causality and are susceptible to confounding by socioeconomic status, overall health, metabolic factors, weight change, and other lifestyle behaviors. Intervention studies demonstrating reductions in epigenetic age often involve small sample sizes, short follow-up periods, or multi-component programs that make it difficult to isolate the specific contribution of individual nutrients or behaviors. Additionally, DNA methylation–based aging signatures are typically measured in peripheral blood, which may not accurately capture tissue-specific aging dynamics in metabolically relevant organs such as liver, adipose, or muscle. Variability across epigenetic clock algorithms, batch effects, and differences in analytical pipelines further contribute to inconsistencies in reported outcomes. Consequently, while current evidence supports biologically plausible links between diet, lifestyle, and epigenetic aging, these associations should be interpreted with caution until validated in larger, rigorously controlled, and mechanistically grounded human studies.

## 7. Multi-Omics and Systems Biology in Personalized Nutrition

**Figure 4:**
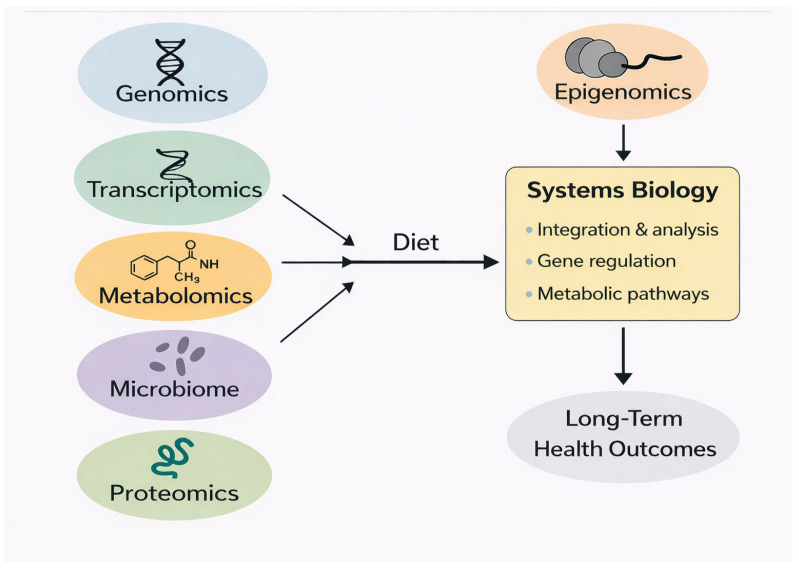
Multi-omics and systems biology framework for personalized nutrition.

Precision nutrition increasingly relies on a systems biology approach that integrates genomics, epigenomics, transcriptomics, metabolomics, proteomics, and microbiome data. Because dietary exposures affect multiple biological layers simultaneously, single-omic measurements provide only a partial view of nutritional responses. Multi-omics integration enables a more comprehensive understanding of how diet influences metabolic pathways, gene regulation, and long-term health outcomes [87,88,89]. Figure 4 provides a schematic overview of how genomics, epigenomics, transcriptomics, metabolomics, proteomics, and microbiome data are integrated through systems biology approaches to inform personalized nutrition strategies.

Epigenomics provides a dynamic readout of environmental influences, while genomics describes inherited predispositions. Transcriptomics captures immediate gene-expression responses to nutrient availability, and metabolomics reflects downstream biochemical alterations in amino acids, lipids, carbohydrates, and microbial metabolites. Microbiome profiling adds additional complexity, as gut microbial composition influences metabolite production, nutrient absorption, immune system, and epigenetic regulation. Short-chain fatty acids, secondary bile acids, and microbial vitamins generated in the gut play important roles in shaping host DNA methylation patterns [90,91]. Concrete applications of multi-omics approach in nutrition research are emerging across several domains. One well-characterized example is the use of combined genomic, microbiome, metabolomic, and clinical data to predict individual postprandial glycemic responses, as demonstrated in large-scale machine learning models where multi-omic integration outperformed carbohydrate-counting or glycemic index–based predictions [92,93]. Another example involves microbiome–epigenome interactions, where short-chain fatty acid (SCFA) production from dietary fiber fermentation has been linked to histone acetylation and altered DNA methylation in colonic epithelial cells, directly influencing inflammatory and metabolic signaling pathways. Multi-omic studies in obesity and metabolic syndrome have also shown that integrating DNA methylation markers with transcriptomic and lipidomic profiles improves prediction of insulin resistance and weight-loss responsiveness compared with single-omic approaches. Additionally, personalized nutrition trials such as Food4Me and related follow-up studies have demonstrated that genotype- and methylation-informed dietary recommendations can better predict changes in body weight, triglycerides, and adherence behaviors than standard population-based guidelines. Collectively, these examples underscore the practical utility of multi-omics integration in advancing precision nutrition.

Systems biology models use machine learning and statistical network approaches to combine these data streams and identify diet-responsive molecular signatures. These models have been used to predict glycemic responses to meals, weight-loss outcomes, lipid metabolism patterns, and inflammation trajectories more accurately than traditional dietary assessment tools [92,93]. Multi-omics–based prediction models often outperform single biomarkers because they capture interactions between genes, environment, and metabolic states.

Integration tools such as weighted gene co-expression networks, pathway enrichment analysis, and multi-omic factor analysis allow researchers to map the molecular pathways that mediate diet–phenotype relationships. Early studies indicate that including DNA methylation in these integrative analyses improves the prediction of dietary responsiveness and helps identify individuals at risk for metabolic disorders [91,92,93]. As computational tools become more advanced, multi-omics–guided nutrition strategies may enable highly individualized dietary recommendations that optimize health outcomes while accounting for genetic background, epigenetic state, microbiome composition, and metabolic phenotype.

## 8. Ethical Considerations and Future Outlook

As nutrigenomics and epigenetic profiling expand into clinical and consumer settings, several ethical considerations must be addressed. The collection and interpretation of genetic and epigenetic data raise concerns related to privacy, data security, informed consent, and potential misuse of personal information. Because DNA methylation is influenced by environment and lifestyle, epigenetic data may reveal sensitive information regarding past exposures, socioeconomic status, or disease risk, necessitating careful handling and transparent data governance practices [51].

Another ethical challenge involves the potential for inequities in access to precision nutrition technologies. Multi-omics testing, advanced sequencing, and AI-driven dietary tools may be costly and inaccessible to low-resource populations, potentially widening health disparities. Ensuring equitable access to personalized nutrition services will require policy frameworks, public health initiatives, and subsidized programs that bridge socioeconomic gaps [90,91].

Interpretation of epigenetic data also poses risks of over-simplification. While DNA methylation patterns correlate with disease risk and biological aging, they are not deterministic. Overemphasis on methylation markers without considering environmental, behavioral, and social factors may promote “epigenetic determinism,” reducing complex biological systems to oversimplified narratives. Ethical communication requires clear explanation of uncertainties, limitations, and the dynamic nature of the epigenome [51].

Regulation of consumer epigenetic testing remains limited. Guidelines are needed to ensure test validity, quality control, responsible marketing practices, and professional oversight. Clinicians and nutrition professionals require training to properly interpret methylation-based biomarkers and avoid unsubstantiated recommendations.

Looking forward, advances in epigenetic clocks, single-cell methylation sequencing, real-time methylation monitoring, and microbiome–epigenome analytics are likely to transform personalized nutrition. Continued innovation will depend on cross-disciplinary collaboration among molecular scientists, clinicians, ethics, data scientists, and public health professionals. The integration of AI, multi-omics, and longitudinal epidemiological studies will enable more precise and predictive nutrition frameworks that support disease prevention, metabolic resilience, and healthy aging [90,91]. Future progress must balance scientific innovation with ethical responsibility, transparency, and equitable access to personalized nutrition technologies.

## 9. Conclusions

While diet is an important modifiable factor influencing the human epigenome, it represents only one component of a broader regulatory landscape that includes genetic variation, developmental programming, cellular metabolism, environmental exposures, and age-related molecular processes. These interconnected influences collectively shape DNA methylation patterns and contribute to interindividual differences in epigenetic regulation. Evidence from molecular studies, clinical interventions, and multi-omics analyses demonstrates that nutrients, bioactive compounds, and dietary patterns can influence DNA methylation dynamics, metabolic pathways, and biological aging. Genetic variation further modifies these responses, underscoring the importance of personalized approaches in nutritional research and practice.

Advancements in epigenetic clocks, high-resolution methylation profiling, microbiome analytics, and systems biology are expanding the potential of precision nutrition. These tools help identify individuals who may benefit from targeted dietary interventions, support early detection of metabolic dysregulation, and offer novel strategies for promoting long-term health. However, significant challenges remain, including the need for standardized methodologies, long-term clinical trials, robust validation of biomarkers, and ethical frameworks that ensure privacy, data security, and equitable access.

Overall, integrating DNA methylation biomarkers with genetic, metabolic, and microbiome data offers a promising pathway toward more effective and individualized nutrition strategies. As research continues to evolve, personalized nutrition informed by epigenetics may contribute to improved disease prevention, optimized metabolic health, and extended health span across diverse populations.

From a practical perspective, the insights summarized in this review highlight several ways in which DNA methylation can be integrated into personalized nutrition and clinical practice. First, methylation biomarkers, particularly epigenetic clocks and nutrient-sensitive CpG sites, may serve as actionable indicators for assessing biological aging, metabolic health, and individual responsiveness to dietary interventions. Such biomarkers could help practitioners tailor nutritional recommendations based on a person’s methylation profile, genetic background, and dietary patterns. Second, the demonstrated influence of methyl donors, polyphenols, and microbiome-derived metabolites on methylation dynamics suggests potential avenues for targeted dietary strategies aimed at improving metabolic resilience, modulating inflammation, and supporting healthy aging. Third, multi-omics and machine-learning frameworks provide an emerging toolkit for predicting personalized dietary responses, enabling more precise and data-driven intervention designs. Finally, incorporating methylation-based insights into public health initiatives may support early identification of at-risk individuals and guide population-level recommendations that consider epigenetic variability. Collectively, these practical applications underscore the growing potential of nutriepigenetics to inform individualized dietary guidelines and enhance long-term health outcomes.

### Advantages and Limitations of Methylation-Based Personalized Nutrition

DNA methylation–guided personalized nutrition offers several advantages, including the ability to capture both genetic predispositions and cumulative lifestyle exposures, providing a dynamic biomarker system that may predict individual dietary responsiveness more effectively than genomic data alone. Epigenetic clocks and nutrient-responsive CpG sites can inform targeted interventions aimed at improving metabolic health, inflammation, and biological aging. However, substantial limitations must be acknowledged. Methylation patterns are highly tissue specific, yet most available clinical measurements rely on peripheral blood, which may not fully reflect organ-specific changes. Inter-individual variability driven by genetics, microbiome composition, and environmental exposures complicates interpretation, and current biomarkers require broader validation across diverse populations. Additionally, methodological differences among methylation assays and ethical considerations related to data security limit immediate translation into routine clinical practice. Thus, while promising, methylation-based personalized nutrition must be applied cautiously and supported by rigorous validation.

## Data Availability

No new data were created or analyzed in this study. Data sharing is not applicable to this article.
